# Impact of gastrointestinal polymerase chain reaction panels on antibiotic utilization in hospitalized adult patients

**DOI:** 10.1017/ash.2023.174

**Published:** 2023-08-02

**Authors:** Gillean A. Kelly, Roumen Iordanov, Alex Franklin, Amna Ahmed, Krithika Srinivasan, Jesica Hayon, Todd Lasco, Rosie Amini, Sabra Shay, Prathit A. Kulkarni, Mayar Al Mohajer

**Affiliations:** 1 Baylor College of Medicine, School of Medicine, Houston, Texas; 2 University of Texas Health Science Center at Houston, School of Public Health, Houston, Texas; 3 Baylor College of Medicine, Department of Medicine, Section of Infectious Diseases, Houston, Texas; 4 Baylor College of Medicine, Department of Pathology & Immunology, Houston, Texas; 5 Premier Inc., Department of Clinical Intelligence, Charlotte, North Carolina; 6 Michael E. DeBakey Veterans Affairs Medical Center, Medical Care Line, Houston, Texas

## Abstract

Multiplex stool polymerase chain reaction (PCR) panels offer rapid comprehensive testing for patients with infectious diarrhea. We compared antibiotic utilization among hospitalized patients with suspected infectious diarrhea who underwent diagnostic testing with either a stool culture or stool PCR panel. No significant differences in antibiotic utilization were identified.

Pathogen identification plays a critical role in determining appropriate therapeutic measures in patients with suspected infectious diarrhea. Routine diagnostic evaluations traditionally include stool culture.^1^ However, evidence suggests that the low proportion of stool cultures that grow pathogenic bacteria^2^ and the lower sensitivity associated with this diagnostic test^3^ limit its clinical utility. Stool cultures are used for bacterial identification and require 24–96 hours for adequate incubation and microbial growth.^3,4^ Importantly, other etiologic agents of infectious diarrhea, such as viruses and parasites, may not be captured by stool culture. Recently, molecular diagnostic tests, such as multiplex polymerase chain reaction (PCR) panels, have emerged as valuable diagnostic tools given their rapid turnaround time, higher test sensitivity, and ability to detect various pathogens.^5^

With the ongoing emergence of drug-resistant organisms, efforts to enhance antimicrobial stewardship are increasingly essential.^6^ Results from stool PCR testing allow clinicians to promptly adjust therapies, offering the potential for judicious and precise antimicrobial use among patients with suspected infectious diarrhea.

We evaluated the impact of multiplex stool PCR testing versus stool culture on the utilization of antibiotics administered for suspected infectious diarrhea in hospitalized adult patients.

## Methods

The study population included hospitalized adult patients at a single quaternary-care center who underwent stool microbiologic testing, either culture or multiplex PCR (the BioFire FilmArray Gastrointestinal Panel, BioFire Diagnostics, Salt Lake City, UT), for suspected infectious diarrhea. Stool cultures were routinely collected from these patients in the preintervention period (October–November 2021). Stool PCR orders were available in that period but were restricted to gastrointestinal and infectious disease providers. All orders for stool culture in the electronic health record (EHR) were converted to a multiplex stool PCR test in December 2021. The postintervention period included January–February 2022; December 2021 was excluded as a washout.

Primary outcomes included antibiotic days of therapy (DOT) and length of therapy (LOT) for the following antibiotics: ceftriaxone, cefepime, ampicillin-sulbactam, piperacillin-tazobactam, amoxicillin-clavulanic, ertapenem, meropenem, metronidazole, ciprofloxacin, levofloxacin, azithromycin. DOT was defined as the summation of days of each antibiotic administered for infectious diarrhea.^7^ LOT was defined as the total number of days of antibiotic therapy for infectious diarrhea, regardless of how many antibiotics were administered per day.^7^ The EHR at our institution is designed such that an indication is required to be selected when ordering antibiotics. When determining DOT and LOT, only ordered antibiotics with an indication for “infectious diarrhea,” “diarrhea,” or “colitis” were included.

To compare variables across the pre- and postintervention groups, the Wilcoxon rank-sum test, the χ^2^ test, and the incidence rate ratio comparison test were used for continuous variables (DOT and LOT), categorical variables (stool positivity), and incidence rate ratios, respectively. R Studio version 4.1.1 software (R Foundation for Statistical Computing, Vienna, Austria) was used for statistical analysis. This study was approved under IRB protocol H-50891 at our institution.

## Results

**Figure 1.**
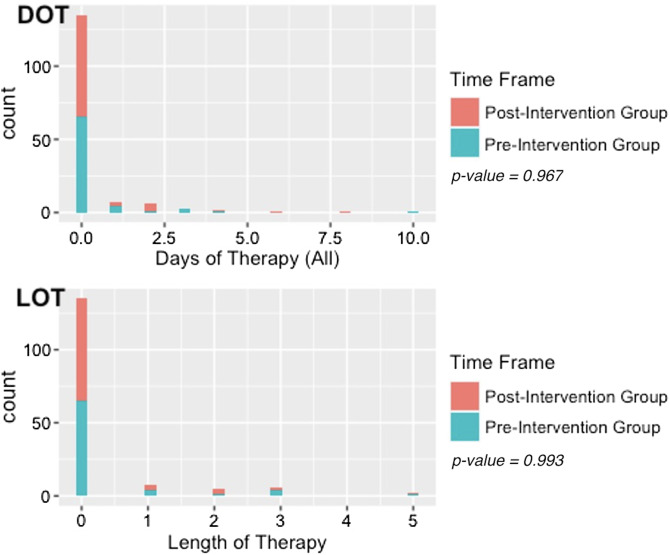
Count of antibiotic days of therapy (DOT) and length of therapy (LOT) by preintervention group (October 2021–November 2021) and postintervention group (January 2022–February 2022).

The pre- and postintervention groups included 75 and 81 patients, respectively. In the preintervention group, stool cultures were negative in 71 (94.7%) of 75 patients, compared to negative stool PCR panels in 58 (71.6%) of 81 patients (*P* < .001) in the postintervention group. In the preintervention group, the median DOT and LOT were both 0 days, with ranges of 0–10 and 0–5 days, respectively. In the postintervention group, the median DOT and LOT were both 0 days, with ranges of 0–8 days and 0–5 days, respectively. There was no statistically significant difference in DOT (*P* = .967) or LOT (*P* = .993) after the intervention (Fig. 1). Similarly, the incidence rate ratios for DOT (0.71; 95% CI, 0.42–1.22) and LOT (0.67; 95% CI, 0.36–1.24) were not statistically different from 1, indicating no change in the incidence rate after the intervention.

## Discussion

Given the rapid and sensitive results available through multiplex PCR,^5^ it may be reasonable to expect a reduction in antibiotic DOT and LOT with stool PCR testing, reflecting a decrease in unnecessary antimicrobials. However, there was no meaningful difference in antibiotic DOT or LOT among hospitalized patients with suspected infectious diarrhea after the introduction of an EHR intervention to switch a stool culture to a stool PCR order. Additionally, there was no remarkable difference in DOT or LOT incidence rates between the groups when adjusted for patient days present.

These findings suggest that using stool PCR panels to replace stool cultures at our institution did not significantly affect antibiotic administration among hospitalized adult patients with suspected infectious diarrhea. Importantly, the proportion of patients in the preintervention cohort who received antibiotics was low (10 of 75, 13.3%) compared to the postintervention cohort (11 of 81, 13.6%). This may have limited our ability to detect a difference in DOT and LOT after introducing the intervention. This lack of initial antibiotic administration may reflect our institution’s existing culture of excellent antimicrobial stewardship. Furthermore, the study’s small sample size (n = 156) might diminish the generalizability of these findings. Additionally, clinical information, such as patient demographics, severity, and presenting symptoms, was not collected.

A study by Singh et al^8^ reflects a similar conclusion. In their retrospective analysis of over 300 patients, they found no significant change in antibiotics between those who received stool PCR (84.9%) compared to stool culture (84.1%).^8^ Notably, among patients who received a stool PCR test, those identified as having a viral infection were observed to have a lower rate of antibiotics than patients with bacterial or parasitic infections.^8^ Although this second observation was not directly measured in our study, the overall conclusions from Singh et al^8^ demonstrated a similar lack of impact on overall antibiotic use with two different stool testing techniques.

However, conclusions from other studies differ from the findings presented here. In a retrospective investigation published in 2019 that included >15,000 patients, stool multiplex PCR panels were associated with significantly lower antibiotic use than traditional stool testing.^9^ By contrast, in a randomized control trial of 74 patients in the emergency department by Meltzer et al,^10^ PCR testing was associated with higher antibiotic utilization compared to traditional or no stool testing among patients with bacterial or parasitic pathogens causing diarrhea. However, the higher antibiotic utilization observed in that particular study likely reflects a rise in appropriate antimicrobial prescriptions informed by PCR detection.^10^

The results of the trial by Meltzer et al^10^ raise another limitation of the generalizability of our study, which is that we investigated total antibiotic usage rather than appropriate antimicrobial prescription. Future initiatives to examine this issue would include expanding the sample size, including multiple hospitals, comparing patient length of stay, and measuring appropriate antibiotic administration compared to unnecessary antimicrobial usage. Additional efforts could incorporate simultaneous interventions, such as formalized education to all providers ordering stool tests and assessing cost comparisons between tests. Future research investigating the impact of stool PCR testing on antimicrobial use may help inform clinical decision making or the development of clinical decision-support tools within EHRs. As antibiotic stewardship becomes increasingly essential with the advent of drug-resistant pathogens,^6^ diagnostic stewardship must be considered in patient evaluation and treatment, including the turnaround time and result sensitivity.
